# Effect of Double Mutation (L452R and E484Q) on the Binding Affinity of Monoclonal Antibodies (mAbs) against the RBD—A Target for Vaccine Development

**DOI:** 10.3390/vaccines11010023

**Published:** 2022-12-22

**Authors:** Deepali Gupta, Mukesh Kumar, Priyanka Sharma, Trishala Mohan, Amresh Prakash, Renu Kumari, Punit Kaur

**Affiliations:** 1 Department of Biophysics, All India Institute of Medical Sciences, New Delhi 110026, India; 2 Division of Bio-Medical Informatics, Indian Council of Medical Research, New Delhi 110029, India; 3 Data Science Division, Amity Institute of Integrative Sciences and Health, Gurgaon 122412, India

**Keywords:** spike protein, double mutation, molecular dynamics, MMGBSA, receptor-binding domain, monoclonal antibodies

## Abstract

The COVID-19 pandemic, caused by SARS-CoV-2, emerges as a global health problem, as the viral genome is evolving rapidly to form several variants. Advancement and progress in the development of effective vaccines and neutralizing monoclonal antibodies are promising to combat viral infections. In the current scenario, several lineages containing “co-mutations” in the receptor-binding domain (RBD) region of the spike (S) protein are imposing new challenges. Co-occurrence of some co-mutations includes delta (L452R/T478K), kappa (L452R/E484Q), and a common mutation in both beta and gamma variants (E484K/N501Y). The effect of co-mutants (L452R/E484Q) on human angiotensin-converting enzyme 2 (hACE2) binding has already been elucidated. Here, for the first time, we investigated the role of these RBD co-mutations (L452R/E484Q) on the binding affinity of mAbs by adopting molecular dynamics (MD) simulation and free-energy binding estimation. The results obtained from our study suggest that the structural and dynamic changes introduced by these co-mutations reduce the binding affinity of the viral S protein to monoclonal antibodies (mAbs). The structural changes imposed by L452R create a charged patch near the interfacial surface that alters the affinity towards mAbs. In E484Q mutation, polar negatively charged E484 helps in the formation of electrostatic interaction, while the neutrally charged Q residue affects the interaction by forming repulsive forces. MD simulations along with molecular mechanics-generalized Born surface area (MMGBSA) studies revealed that the REGN 10933, BD-368-2, and S2M11 complexes have reduced binding affinity towards the double-mutant RBD. This indicates that their mutant (MT) structures have a stronger ability to escape from most antibodies than the wild type (WT). However, EY6A Ab showed higher affinity towards the double MT-RBD complex as compared to the WT. However, no significant effect of the per-residue contribution of double-mutated residues was observed, as this mAb does not interact with the region harboring L452 and E484 residues.

## 1. Introduction

The ongoing coronavirus pandemic (COVID-19) has affected lives across the globe. Vaccination and neutralizing antibodies (NAbs) have emerged as the most promising strategies to end the pandemic [1]. The emergence of more virulent variants of SARS-CoV-2 has triggered intensive genomic surveillance [2]. S glycoprotein (S) of the viral genome mediates the fusion of viral proteins with the human angiotensin-converting enzyme 2 (hACE2) receptor [3]. Therefore, S glycoprotein is considered the primary target for most vaccine and therapeutic antibodies [3,4]. The rapidly spreading lineage B.1.617.1 was called the kappa variant of interest (VOI) by the World Health Organization (WHO) [5]. It was first identified in India in December 2020 and has spread across the globe to at least 60 countries. This variant harbors co-occurrent key mutations L452R and E484Q in the RBD of the S protein. The kappa variant shares the L452R mutations with B.1.427/429 (epsilon) and the E484Q mutation of the beta (B.1.351) and gamma (P.1) lineages, where Residue 484 is mutated to lysine (E484K) [6]. The dominant Omicron variant, which was first detected in November 2021 in Southern Africa, quickly spread to over 60 countries [7]. The high transmission rate and short doubling time soon made it the dominant virus. The S protein of the Omicron variant has been reported to possess more than 30 mutations [8]. Out of these, 16 mutations occur in the RBD of the S protein, making it the ideal target for neutralizing antibodies and immunotherapies. The most prevalent RBD mutations include G339D, S371L, S373P, S375F, K417N, N440K, G446S, S477N, T478K, E484A, Q493R, G496S, Q498R, N501Y, and Y505H [9]. One interesting mutation, L452R, initially absent in the Omicron variant, was later reported in 25.9% of cases as per GISAID data on 5 December [10]. The L452R mutation was observed to be linked with high fusogenicity, infectivity, and glycolysis in pseudoviruses [11].

Two mutations (L452R, E484Q), reported in other SARS-CoV-2 variants, have been found to be associated with immune escape and increased transmissibility and pathogenicity. Evidence supports that the L452R mutation is present in the B.1.429 variant [12], while the E484Q mutation is observed in the B.1.1.7, B.1.351, P.1, and B.1.526 variants [13,14]. The L452R mutation has been shown to enhance viral infectivity and transmissibility [15]. E484Q, along with L452R substitution, has been found to be functionally related to hACE2 binding and immune evasion [16].

The trimeric S protein harbors the receptor-binding domain (RBD), which provides conformation plasticity to interact with the human angiotensin-converting enzyme 2 (hACE2) receptor [17]. The RBD can exist in three conformations, and previous reports have revealed that it can switch between three states: open (up), closed (down), and intermediate (one RBD up or two RBD up) while attaching to the hACE2 receptor. The RBD is the prime target for various neutralizing mAbs in both humoral and cell-mediated immunity.

Two co-occurring mutation residues (L452, E484) are located near the hACE2 binding site of the RBD, which may influence the binding affinity of ACE2 and the neutralizing activity of mAbs. Based on epitope recognition and binding, mAbs are grouped into four classes (I to IV) [18]. Cryo-EM structures of the different classes of mAbs are available [19]. Class I mAbs overlap with the hACE2 receptor site and bind to the open conformation of the RBD (BRII-196, S2E12, CC12.1, REGN10933, BD-236). Class II mAbs bind to both open and closed RBD conformations at different epitope sites (P2B-2F6, BD-368-2, C002, C121, LY-CoV555, and C119). Class III mAbs target both up and down RBD conformations but bind outside the hACE2 binding site (S2M11, C144, BD-23, 2-4). Class IV mAbs do not overlap with the hACE2 binding site but interact with the open state of the RBD situated outside the RBM region (S309, C135, REGN10987, 2-43, EY6A, S2A4, CR3022, and S304) [20]. Class IV antibodies exhibit weak neutralizing activity. Recently, a new Class V mAb (JMB2002) has been shown to have anti-neutralizing activity against the Omicron variant [21]. Due to its efficacy and pharmacokinetic properties, this mAb has entered Phase I clinical trials in the USA (IND 154745) [22].

Two mutations (L452R and E484Q) that occur consistently in the kappa variants are localized in and near the binding surface between the RBD and hACE2 and have been found to be associated with immune escape. It is promising that the Class IV mAb, recognized as a different epitope away from the double-mutant site, has poor neutralizing activities [23].

**Figure 1:**
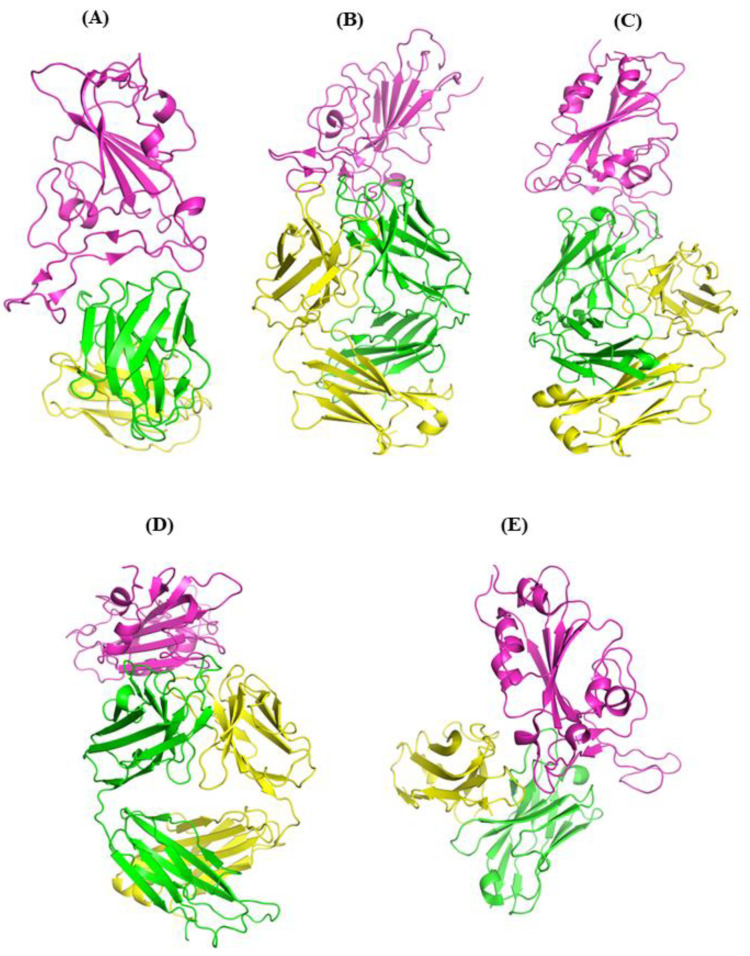
Cartoon representation of crystal structures of spike protein RBD in complex with monoclonal antibodies (mAbs): (**A**) REGN10933 [25] (PDB ID: 6xdg), (**B**) BD368-2 [26] (PDB ID: 7chf), (**C**) S2M11 [27] (PDB ID: 7k43), (**D**) EY6A [28] (PDB ID: 6zcz), and (**E**) JMB2002 [21] (PDB ID: 7wrv). RBD is shown in magenta, antibody heavy chain (HC) in green, and antibody light chain (LC) in yellow.

The effect of double mutations (L452R and E484Q) on the binding capability of hACE2 binding has already been studied [24]. These two mutations affect the microenvironment of the interfacial region where the RBD and hACE2 interact to enhance the overall binding stability of the bound complex. This double mutation not only enhances the binding of the RBD with hACE2 but is also a target for neutralizing antibodies. In the present study, the molecular mechanisms behind the impact of these mutations on the binding affinity of the RBD with each member of mAbs were investigated through MD simulations combined with the free-energy calculations method. Atomic structures of several mAbs in complex with the RBD of the S protein of SARS-CoV-2 are available in the PDB. A representative Ab complex for each class was considered in this study. A representative Ab complex for each class—REGN10933 for Class I [25], BD-388-2 for Class II [26], S2M11 for Class III [27], EY6A for Class IV [28], and JMB2002 [21] for Class V—was selected (Figure 1). MD simulations were performed for the Ab complex of each class to study the effect of the double mutation on antibody binding. This work contributes to understanding the molecular interaction between the viral RBD and different monoclonal antibodies (mAbs). This can aid in developing new drugs and vaccine strategies in the future, as well as in ascertaining the efficacy of existing drug therapy.

## 2. Materials and Methods

### 2.1. Preparation of Wild-Type and Mutated Ag-Ab Complex

The three-dimensional coordinates of structures of the wild-type (WT) RBD bound to monoclonal antibodies (mAbs) were retrieved from the Protein Data Bank (Table 1).

The structures were prepared using the Protein Preparation Wizard by assigning the correct bond order and ionization states; adding missing atoms/side chains; assigning partial charges; and eliminating unwanted sugar molecules, cofactors, and ligand molecules [29]. In all the four complexed structures, the missing loop regions of the RBD were built using Schrödinger Prime [30]. Hydrogen atoms were added, and a standard protonation state at pH 7.0 was used [31]. The co-mutant complexes in Classes I to V were generated by mutating the amino acids at Position 452 from leucine (L) to arginine and replacing glutamic acid (E) with glutamine (Q) at Position 484. These mutant complexes (MT) were similarly processed using the Protein Preparation Wizard. For the Class V mAb, the interactions of the Omicron-mutated RBD with the mAb were obtained using the Pymol and PDBsum server.

MD simulations were implemented using the Desmond module. The OPLS force field parameter was employed to run all calculations [32]. Both wild-type (WT) and mutant-type (MT) RBD structures, along with the mAb complex, were positioned in an orthorhombic box that was solvated with the TIP3P water system [33]. This step was performed using the System Builder menu of Desmond. Sodium and chloride ions were added to neutralize the system. Both WT and MT-RBD complexes, along with their respective mAbs, were subjected to Desmond’s default eight-stage relaxation protocols before the commencement of the production run. All simulations were carried out in an isothermal–isobaric ensemble (NPT) system. For the simulations, the Nose–Hoover thermostat and the isotropic Martyna–Tobias–Klein barostat were used to maintain the pressure at 1 atm and temperature at 300 K. The cut-off distance for short-range interaction was set at 10.0 Å, while the long-range columbic interactions were assessed using the smooth particle mesh Ewald method (PME). A time-reversible reference system propagator algorithm (RESPA) integrator was used to accelerate the run, with an inner time step of 2.0 fs. The MT-RBD complexed structure with mAbs was extracted at 100 ns of MD simulations and was superimposed with their respective WT-RBD structures to identify the conformation changes using PYMOL.

### 2.2. Trajectory Analysis after Molecular Dynamics (MD) Simulation

The root mean square deviation (RSMD) represents the deviation from the initial minimized crystal structure for each of the system studies, while root mean square fluctuation (RMSF) represents the overall trend of flexibility of each residue in the protein. The change in RMSD values indicates the local conformational alternations occurring in the backbone of the protein residues due to these mutations. A higher RMSF value indicates increased flexibility, which points towards their potential to interact with other molecules, while a lower RMSF indicates lower fluctuation, implying less flexibility and diminished binding potential. RMSD and RMSF values for the protein backbone were taken using the simulation interaction diagram tool implemented in Desmond, and a graph was generated for the same values. The molecular interactions of the S protein RBD with mAbs were determined by submitting the obtained PDB at the PDBsum server [34].

### 2.3. MMGBSA Analysis

Binding free energies of the RBD complex with different mAbs were calculated using Schrödinger Prime employing the VSGB 2.0 solvation model [35]. For each RBD-mAb complex, energy was computed by taking 200 frames from the last 20 ns of simulation time [36]. The MMGBSA analysis calculates the binding energy and its constituent individual energy terms, including columbic, covalent, van der Waals (vdW), lipophilic (lipo), generalized Born electrostatic solvation (Solv GB), and hydrogen bonding (H bond) terms [37]. The obtained MMGBSA energy values were then averaged, and the standard deviation was calculated. Herein, the net binding free energy was computed using the thermal_mmgbsa.py script.

Per-residue decomposition was obtained, to identify the energy contribution of the crucial RBD residue that participated in effective binding with mAbs. The per-residue interaction energy was calculated using breakdown_MMGBSA_by_residue.py.

## 3. Results and Discussion

### 3.1. Comparisons of Biomolecular Interaction between the mAb and RBD Complexes

The molecular interactions between the SARS-CoV-2 RBD and mAb were studied after 100 ns MD simulation.

#### 3.1.1. Interaction of REGN10933 with WT and MT-RBD

**Figure 2:**
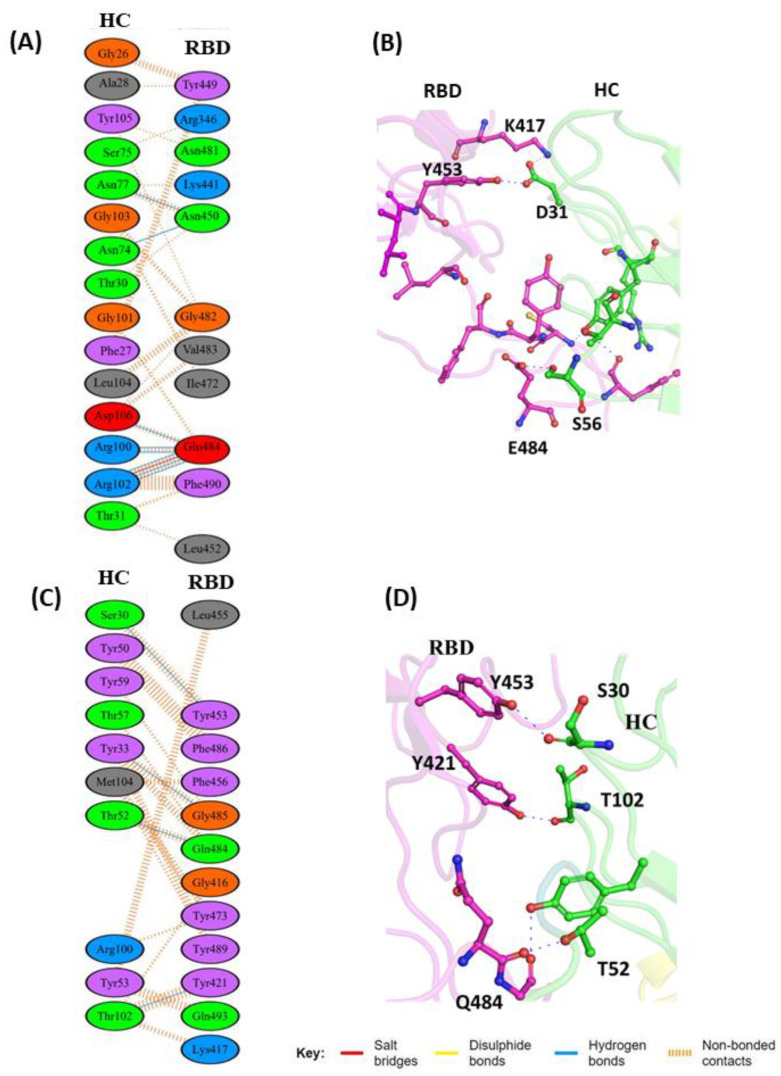
Protein–protein interaction between RBD with REGN10933 antibody: (**A**) 2D interaction diagram of binding interface of WT-RBD with REGN10933 antibody; (**B**) 3D cartoon representation of intermolecular interaction of E484 residue of RBD with heavy chain (HC) of REGN10933 antibody; (**C**) 2D interaction diagram of binding surface of MT-RBD with REGN10933 antibody; (**D**) 3D cartoon representation of intermolecular interaction of E484 residue of RBD with heavy chain of REGN10933 antibody. Here, only intermolecular interactions are shown. RBD represents receptor-binding domain, while HC represents heavy chain of monoclonal antibody (mAb). The interacting residues are shown in sticks. RBD is shown in magenta cartoon, and HC is shown in green color as a residue of mAbs (Figure 2B). In the MT-RBD-REGN1093 complex, the replacement of the hydrophobic Leu (L) residue with positively charged polar Arg (R) residue abolishes the intramolecular hydrophobic contact with L490 (Figure S1A,B), while the substitution of Glu (E484), which has a polar acidic side chain with Gln (Q), results in the formation of a new hydrogen bond interaction with T52 and Y33 residues of HC. The key salt bridge interaction of K417 in RBD with D31 (HC region) was found to be lost in the MT-RBD complex (Figure 2C,D). The WT-RBD complex with REGN10933 displayed additional stabilizing interactions as compared to the MT complex.

The cryo-electron microscopy (cryo-EM) [38] structure of the Fab region of REGN10933 reveals that it binds to the RBD from the topmost site and overlaps with the hACE2 binding site [38]. We studied the effect of the RBD double mutation on mAb binding by comparing the molecular interactions between the wild-type (WT) and mutated-type (MT) RBD complexes. The interfacial residues between the RBD and REGN10933 were analyzed in the post-MD complexes (Figure 2A,B). Several hydrogen bonds present between the heavy-chain (HC), complementarity-determining regions (CDRs) and the RBD, involving the HC residues D31, Y33, and T52 and the RBD residues K417, Y453, E484, F486, C488, and Y489 (Figure 2A). The complementarity-determining regions (CDRs) are the hypervariable domains of the Ab that determine the specific antibody interaction with the antigen molecule. The light-chain (LC) residues of the mAb are responsible for stabilizing the HC residues to facilitate the interaction with the RBD of the S protein. Here, we focused specifically on the co-mutant residues of the RBD, i.e., L452 and E484, and their induced local perturbations on the protein’s overall structure. The L452 (RBD) residue made key intramolecular hydrophobic interactions with L492 and F490 that constitute the hydrophobic patch on the surface of the RBD. The E484 residue forms intermolecular interactions with the HC of REGN10933 through the S56.

#### 3.1.2. Interaction of BD-368-2 with WT and MT-RBD

**Figure 3:**
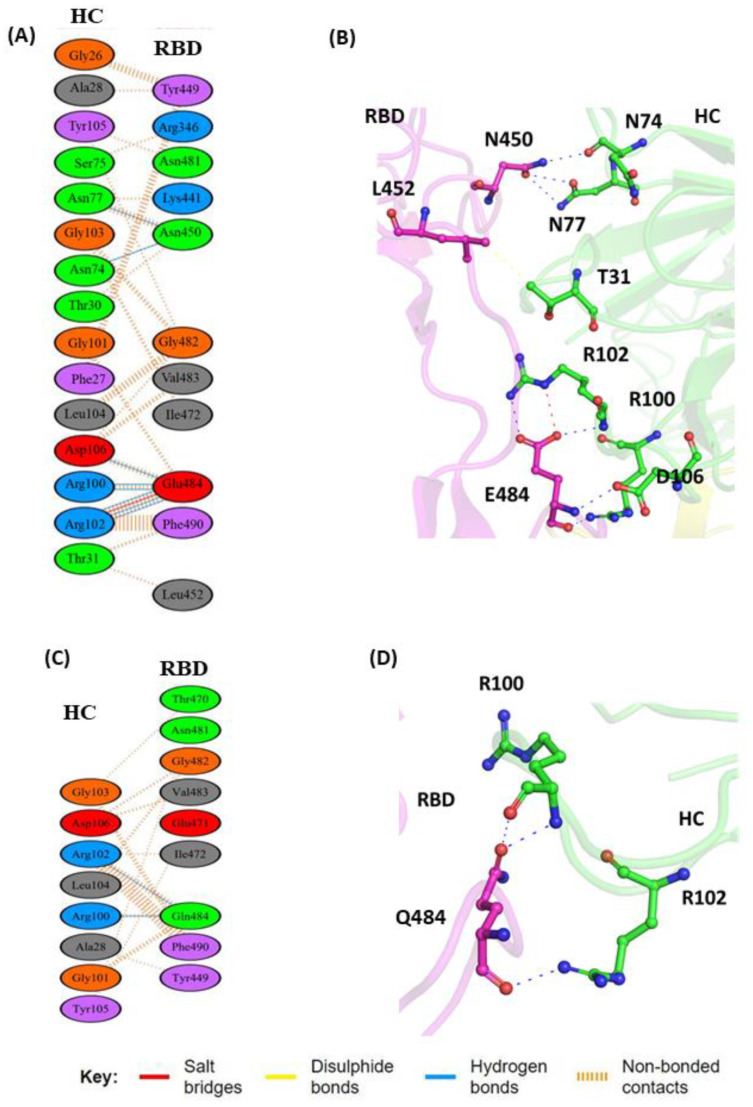
Protein–protein interaction between RBD and BD-368-2 monoclonal antibody: (**A**) 2D interaction diagram of binding interface of WT-RBD with BD-368-2 antibody; (**B**) 3D cartoon representation of intermolecular interaction of L452 and E484 residue of RBD with HC of BD-368-2 antibody; (**C**) 2D interaction diagram of binding surface of MT-RBD with BD-368-2 antibody; (**D**) 3D cartoon representation of intermolecular interaction of E484Q and L452R residue of RBD with HC of BD-368-2 antibody. The interacting residues are shown in sticks. Here, only intermolecular interactions are represented. RBD represents receptor-binding domain, and HC represents heavy chain of monoclonal antibody.

BD-368-2 is one of the most potent neutralizing monoclonal Abs against SARS-CoV-2 and gave an IC50 of 1.2 and 15 ng/mL against both pseudotyped and authentic virus, respectively [39]. This mAb also exhibited high therapeutic and prophylactic efficacy in human ACE2 (hACE2) transgenic mice infected by SARS-CoV-2 [39]. BD-368-2 fully blocks the hACE2 recognition and targets both the “up” and “down” RBD conformations. The result showcased that the WT-RBD complex with the mAb complex is stabilized by multiple hydrogen bonds, salt bridge interactions, and non-bonded contacts as compared to its mutant type (Figure 3A,B). Three Fab regions of heavy-chain BD-368-2 (CDRH1, CDRH3, and DE loop in the VH domain) are involved in interaction with the RBD [26]. However, in CDRH1, the residues G26, F27, and A28 form a prominent non-covalent interaction with the residue Y449 (RBD). The residue N450 of the RBD forms hydrogen bond interactions with N74 and N77 of the HC. The amino acid residue L452 is involved in intermolecular contacts and interacted through non-covalent contacts with T31 of the HC (mAb) (Figure 3A). E484 (RBD) was observed to be the key residue that facilitated both salt bridge and hydrogen bond interactions with the R100 (HC), R102 (HC), and D106 (HC) residues (Figure 3A,B). The exchange of the non-polar leucine residue at Position 452 with a hydrophilic, polar arginine residue (L452R mutation) sterically restricted the interaction of L452 with the T31 residue of the HC. This interaction also influenced the hydrogen bond interactions formed by N450 (RBD). The substitution E to Q in the mutant resulted in a slight movement of N450 (3.5 Å) away from the HC of the mAb, leading to the disruption of this interaction (Figure 3C). The salt bridge that formed between E484 (RBD) and R102 (HC) at the interfacial binding region was also not observed due to altered polarity and different spatial conformation imposed by the uncharged Gln residue (Figure 3D).

#### 3.1.3. Interaction of S2M11 with WT and MT-RBD

**Figure 4:**
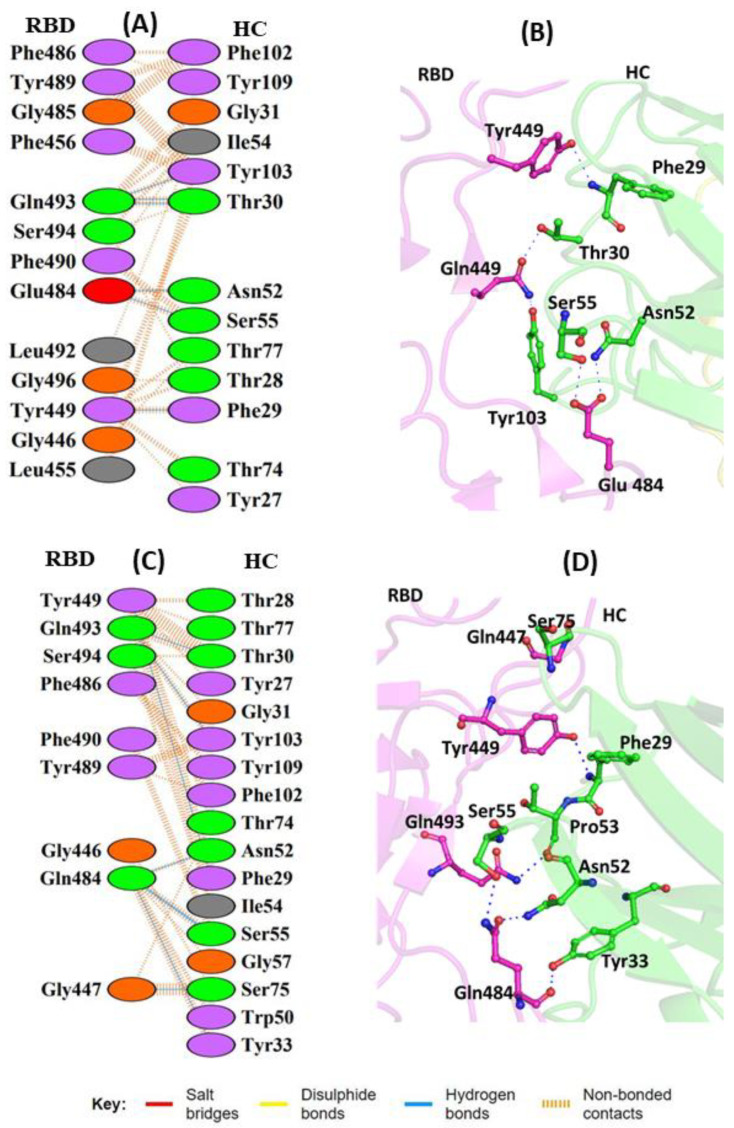
Protein–protein interaction between the RBD and S2M11 monoclonal antibody: (**A**) 2D interaction diagram of binding interface of WT-RBD with S2M11antibody; (**B**) 3D cartoon representation of intermolecular interaction of E484 residue of RBD with HC of S2M11 antibody; (**C**) 2D interaction diagram of binding surface of MT-RBD with S2M11 antibody; (**D**) 3D cartoon representation of intermolecular interaction of E484Q residue of RBD with HC of S2M11 antibody. The interacting residues are shown in sticks. Only intermolecular interactions are indicated. RBD represents receptor-binding domain, and HC represents heavy chain of monoclonal antibody.

S2M11 belongs to Class III mAbs that bind away from the hACE2 binding region. These mAbs possess the ability to bind the RBD in both up and down conformation. The S2M11 mAb associates with the RBD to form a bridge between the neighboring RBDs present on the S trimer surface [40]. This mAb has the ability to fully block hACE2 binding by interacting with the two adjacent or all three RBDs simultaneously. S2M11 is known to exhibit strong therapeutic and prophylactic effects against both WT and pseudotyped (PT) virus [41]. This mAb targets the two neighboring RBDs of the same trimer consisting of the receptor-binding motif (RBM) residues from one S monomeric unit and the RBM from an adjacent S monomeric unit. Most of the WT-RBD interactions are mediated through heavy-chain CDRs (HCCDRs) of S2M11. The L452 residue does not interact with the mAb molecule; however, it mediates the formation of the intramolecular hydrophobic patch with F490 and L492. The substitution of hydrophobic L452 with the positively charged R residues abolishes the hydrophobic interaction with L492. The side chains of residues Y449 and E484 of the RBD are hydrogen-bonded to the F29 backbone amide and the N52/S55 side chains of S2M11 heavy chain, respectively (Figure 4A,B). Q493 makes hydrogen-bonded interactions with both T30 and Y103 of the heavy-chain CDR of S2M11 (Figure 4A). The replacement of E484 (RBD) with the negatively charged Q residue retained the original interaction with N52 and S55 of the HC residues and resulted in the formation of an additional hydrogen bond with Y33 (HC) (Figure 4C,D). In the MT-RBD complex, an extra hydrogen-bonded interaction was observed in comparison to the WT complex. However, the WT complex displayed the presence of a higher number of non-bonded contacts.

#### 3.1.4. Interaction of EY6A with WT and MT-RBD

**Figure 5:**
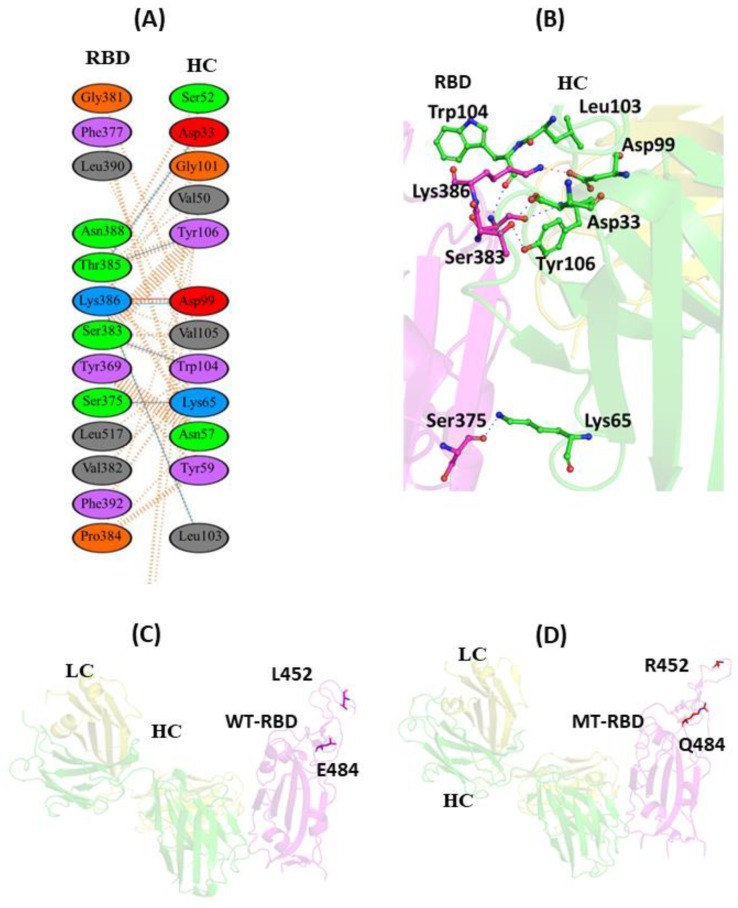
Protein–protein interaction of RBD with EY6A mAb: (**A**) 2D interaction diagram of binding interface of WT-RBD with EY6A antibody; (**B**) 3D cartoon representation of intermolecular interaction of E484 residue of RBD with HC of EY6A antibody; (**C**) cartoon representation of WT-RBD with EY6A antibody. The residues L452 and E484 are represented in sticks. These residues lie far away from EY6A binding site and do not participate in forming intermolecular contact with mAb; (**D**) Cartoon representation of MT-RBD with EY6A mAb. R452 and Q484 residues of RBD are shown in sticks. These residues also do not interact with EY6A antibody. RBD represents receptor-binding domain, HC represents heavy chain of monoclonal antibody, while LC represents light chain of mAb.

The Fab region of EY6A binds at the RBD site, which is completely distinct from the hACE2 binding site. Previous experiments with surface plasmon resonance (SPR) revealed that the Fab fragment of EY6A binds to immobilized SARS-CoV-2 RBD with a *K*D value of 2 nM [42]. EY6A binds to the RBD at the α2-helix (Residues 365–371) and the α3-helix (Residues 384–388) away from the loop region where the residues L452 and E484 are located. A total of 12 residues from the HC and 13 residues from the RBD participate to form the binding interphase. The residue T385 of the RBD interacts with G101 and Y106 residues of the HC through hydrogen bond interaction. The residues K386 (RBD) and D99 (HC) are involved in a salt bridge interaction and further stabilize the complex. Y106 residue of the HC also forms hydrogen bond contacts with T385 of the RBD. However, six hydrogen bond interactions along with other non-bonded hydrophobic interactions further increase the binding affinity of EY6A with the RBD (Figure 5A,B). Since the residues L452 and E484 do not directly make any contact when EY6A binds to the RBD, the effect of these mutations on EY6A binding was not observed (Figure 5C,D).

#### 3.1.5. Interaction of JMB2002 with WT and MT Omicron-RBD

**Figure 6:**
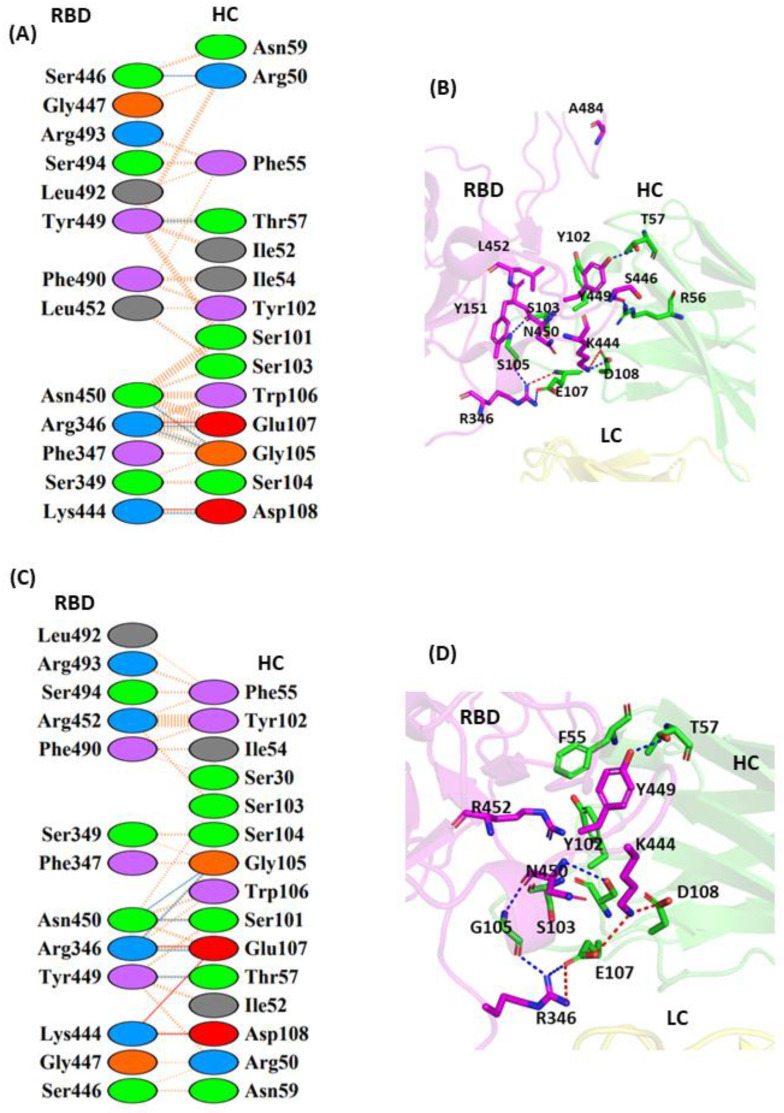
Protein–protein interaction between the RBD and JMB-2002 monoclonal antibody: (**A**) 2D interaction diagram of binding interface of WT-RBD with JMB-2002 antibody; (**B**) 3D cartoon representation of intermolecular interaction of L452 residue of RBD with HC of JMB2002 antibody; (**C**) 2D interaction diagram of binding surface of L452R-RBD with JMB2002 antibody; (**D**) 3D cartoon representation of intermolecular interaction of L452R residue of RBD with HC of JMB2002 antibody. The interacting residues are shown in sticks. Only intermolecular interactions are indicated. RBD represents receptor-binding domain, HC represents heavy chain of monoclonal antibody, and LC represents light chain of monoclonal antibody.

JMB2002 represents a new class of SARS-CoV-2 neutralizing antibody that has a different mechanism of binding from other reported Nabs, and hence has been classified as a Class V Nab. The reported electron microscopy structure revealed that the Fab region of JMB2002 binds only to the down conformation of the RBD and inhibits hACE2 binding. The residues R346 and K444 of the RBD dually interact with E107 and D108 of mAbs HC, respectively, to form both a salt bridge and a hydrogen bond (Figure 6A,B). The complex is further stabilized through a hydrogen bond interaction with the S446 (RBD)/R50 (HC) and Y449 (RBD)/T57 (HC) residues. The L452 residue situated in the middle of the binding epitope forms a hydrophobic interaction with Y102 and S103 of the HC of Nab. The E484A mutation in Omicron helps to improve the interaction between the RBD and hACE2 and displays strong affinity towards hACE2. The residue A484 in Omicron is observed to lie away from the binding epitope of JMB2002 (Figure 6A). In the L452R MT Omicron–JMB2002 complex, as compared to the WT, an additional salt bridge interaction is observed between K444 (RBD) and E107 (HC) that is responsible for the enhanced affinity of the RBD against this Nab. R452 residue in the mutated RBD complex forms hydrophobic contacts with the F55, Y102, and S103 residues of the HC. The predominance of non-polar residues can result in hydrophobic clash of this Nab with the RBD (Figure 6C,D).

### 3.2. RMSD Calculations

**Figure 7:**
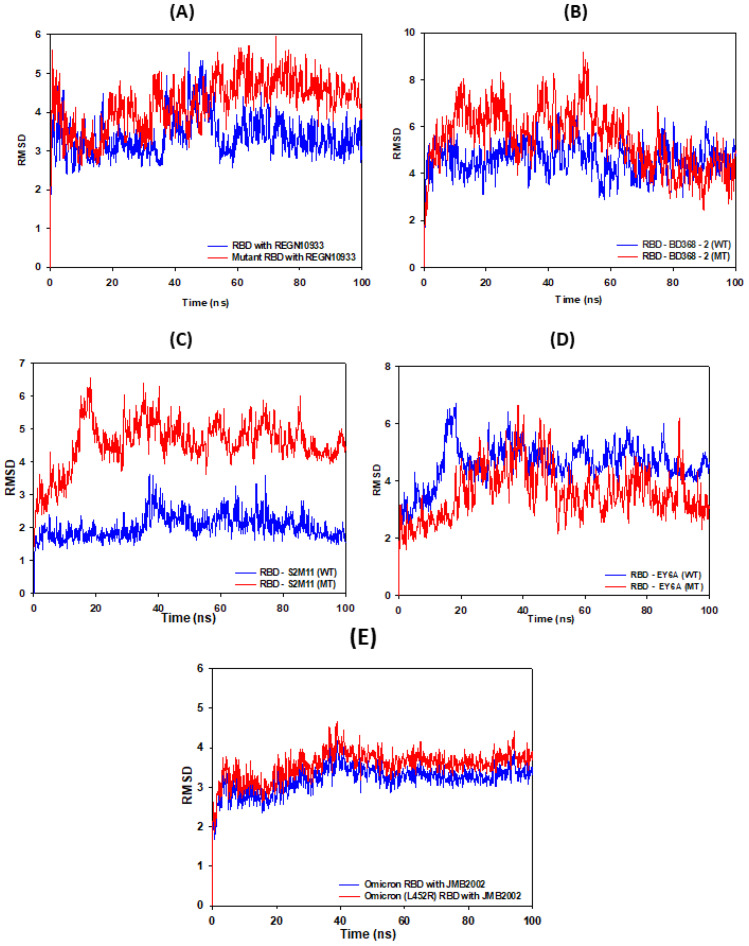
The root mean square deviation (RMSD) of wild-type (WT) and mutant-type (MT) RBD in presence of (**A**) REGN10933, (**B**) BD368-2, (**C**) S2M11, (**D**) EY6A, and (**E**) JMB2002 mAbs during 100 ns MD simulation run.

RMSD comparison was undertaken by superimposing the obtained snapshots on their respective crystal structures. The RBD, in contrast to mAb, is a somewhat rigid ensemble due to the occurrence of numerous β pleated sheets structures. RMSD plots of all complexes gave the mean RMSD values for the WT-RBD complex with REGN10933, BD368-2, S2M11, and EY6A as 4.0 ± 0.5 Å (Figure 7A), 5.0 ± 0.6 Å (Figure 7B), 3.0 ± 0.3 Å (Figure 7C), 5.2 ± 0.5 Å (Figure 7D), and 3.7 ± 0.5 Å (Figure 7E), respectively, while mutant complexes were found to be 5.0 ± 0.5 Å (Figure 7A), 6.0 ± 0.5 Å (Figure 7B), 6.0 ± 0.5 Å (Figure 7C), 5.4 ± 0.5 Å (Figure 7D), and 3.7 ± 0.5 Å (Figure 7E), respectively.

### 3.3. RMSF Calculations

**Figure 8:**
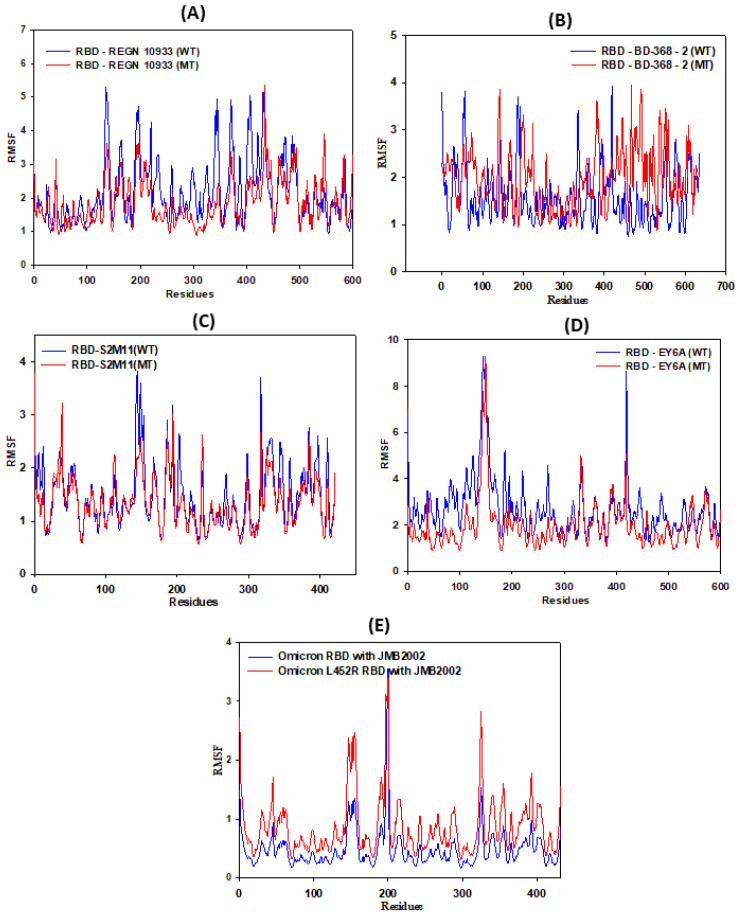
The root mean square fluctuation (RMSF) of wild-type (WT) and mutant-type (MT) RBD in presence of (**A**) REGN10933, (**B**) BD368-2, (**C**) S2M11, (**D**) EY6A, and (**E**) JMB-2002 mAbs during 100 ns MD simulation run.

The RBD consists of both β sheets and a few loop regions, while the HC and LC regions of mAbs mostly contain random coil structures. The higher fluctuations of both WT and MT complexes are mainly due to the presence of the loop regions. MT-RBDs of REGN10933, BD-368-2, S2M11, EY6A, and JMB-2002 display relatively more fluctuation as compared to their WT complexes (Figure 8). The values for the WT and MT-L452R/E484Q residue have been provided in Table 2. Analysis of RMSF values of L452R and E484Q revealed a greater degree of fluctuation in the mutant structure as compared to WT.

### 3.4. Gibbs Free-Energy Calculations for WT and MT-RBD Complex with mAbs

The binding affinity of mAbs with the RBD was further evaluated through MM/GBSA calculations for the 200 conformations observed in the last 20 ns of the MD simulation run. Our result indicates that the WT complexes of the RBD with REGN10933 (−87.58 ± 3.2), BD-362-2 (−65.43 ± 1.2), S2M11 (−78.68 ± 3.2), and JMB2002 (−82.68 ± 4.2) possess high thermodynamic stability as compared to their MT complex, REGN10933 (−76.29 ± 1.7), BD-362-2 (−17.96 ± 1.2), S2M11 (−60.47 ± 1.2), and JMB2002 (−76.28 ± 3.2), respectively (Table 3). However, the MT-RBD-EY6A (−96.47 ± 6.2) was found to be more stable than its WT (−88.23 ± 0.87) complex.

The WT-RBD complex with the REGN10933 mAb possesses favorable columbic contribution energies (−40.66 ± 2.5) as compared to its respective MT complex (−29.528 ± 1.2). This might occur due to the salt bridge interaction formed between residues K417 of the RBD and D31 of the HC of mAbs (Table 3). The WT-RBD complex with BD-362-2 forms a higher number of interactions with contribution by an additional salt bridge, which imparts it higher hydrogen bond and van der Waals interactions, contributing to its higher columbic (−89.94 ± 3.2), hydrogen bond (−5.3 ± 0.05), and van der Waals interaction energy (−98.01 ± 6.7) over the MT complex (columbic (−17.96 ± 1.2), H bond (−5.3 ± 0.05), and van der Waals interaction energy (−10.52 ± 0.52) (Table 3). Similarly, the WT-RBD complex of S2M11 exhibited higher negative ΔGbind columbic (−39.58 ± 5.2), covalent (−6.51 ± 0.65), hydrogen bond (−1.85 ± 3.2), and van der Waals (Vdw) interaction energy (−33.27 ± 0.04) over the MT complex (columbic = −49.03 ± 3.2, covalent = −2.27 ± 0.39, H bond = −1.8 ± 0.09) and van der Waals interaction energy (= −90.3 ± 3.2), imparting it higher binding affinity (Table 3). In the case of the three mAbs, it can be predicted that the mutation significantly lowers the binding energies between the RBD and mAb, which may weaken the neutralization activity of these antibodies leading to evasion of immune response. However, in Class IV mAb, the MT-RBD-EY6A complex displayed higher binding energy (ΔGbind −96.23 ± 0.87) in relation to its WT complex (−88.23 ± 0.87) (Table 3). This can be attributed to comparatively high electrostatic (ΔG) Coulomb (−74.42 ± 3.5) interactions in the MT-RBD complex as compared to the WT complex. These mAbs may retain their neutralization activity against the mutated virus. Along with non-polar interactions, Omicron-RBD forms several hydrogen bonds and two salt bridge interactions with JMB2002 that contribute to its higher overall stability as compared to the L452R mutant. The interaction energies (Columbic (−96.23 ± 1.2), H bond (−5.53 ± 0.05) and van der Waals interaction energy (−78.52 ± 0.52) contribute to higher negative ΔGbind (−82.68 ± 4.2) and in turn higher binding affinity. In L452R-mutant Omicron, one extra salt bridge imparts it more negative columbic energy (−106.53 ± 6.2). However, due to the destabilizing effect of the L452R mutation, the overall binding energy is computed to be lesser in comparison to Omicron-RBD (−76.28 ± 3.2). It can be hypothesized that JMB2002 may have lower neutralization activity against L452R mutant as compared to Omicron.

### 3.5. Per-Residue Energy Contribution

Per-residue decomposition analysis was performed to estimate the energy contribution of the single residues of the RBD by summing its interactions over all HC and LC residues in the complex. In the WT-RBD-REGN10933 complex, the ΔGbind contribution of L452 (−1.36 kcal/mol) and E484 (−4.93 kcal/mol) residues was higher as compared to the residues in the MT complex (L452R (−0.36 kcal/mol) and E484Q (0.51 kcal/mol) (Table 4). This is due to the more negative columbic, van der Waals, and hydrogen bonding interactions observed as compared to the MT complex. The L452R mutation constitutes the replacement of non-polar Leu residue by the polar Arg, which contributes to the formation of interactions with solvent molecules as well as to the overall stabilization of the complex. The ΔGbind contribution clearly points towards the formation of more hydrogen bond and non-bonded contact in the WT complex over the MT complex, leading to a stronger binding ability to the Ab (Table 4). In WT-RBD-BD-6322, the energy contribution of L452 (−1.17 kcal/mol) and E484 (−5.37 kcal/mol) residues was found to be comparatively higher than its respective MT complex ((L452R (−0.87 kcal/mol) and E484Q (−0.90 kcal/mol)) (Table 4). The L452 residue of the RBD forms intermolecular van der Waals contacts with T31 (HC) of BD-6322, contributing to its more negative van der Waals energy (ΔG Vdw −1.54 kcal/mol) over the MT-RBD-BD-6322 complex (ΔG Vdw −0.54 kcal/mol). The columbic and hydrogen bond energy contribution of E484 residue was found to be higher for the WT-RBD-BD-6322 complex as compared to MT-RBD-BD-6322 (Table 4). This can be accounted for by the strong salt bridge interaction and more hydrogen bond interaction in the WT complex as compared to the MT complex (Figure 4). The energy contribution for the residue L452 (−2.57 kcal/mol) in WT-RBD-S2M11 was comparatively more negative than its respective mutant complex (−0.86 kcal/mol). The increased energy is associated with favorable intramolecular van der Waals energy in WT-RBD over MT-RBD. Q484 has more ΔG H bond (−0.33 kcal/mol) as compared to E484 (−0.21 kcal/mol), which is further complemented with an additional hydrogen bond observed in WT (Table 4). The EY6A mAb does not present any interaction with L452 and E484 residues of the RBD. No significant change in per-residue binding energy contribution of co-mutant L452R and E484Q was observed, as this mAb binds at a region situated away from the hACE2 binding site (Table 4). Since it does not bind at the hACE2 binding site, EY6A mAb can be used in combination with other neutralizing nanobodies to provide a blueprint for designing antibody cocktails and therapeutics. In Omicron-RBD, the ΔGbind contribution of L452 (−2.19 kcal/mol) residues was higher as compared to its L452R complex (L452R (−0.76 kcal/mol) (Table 4). The more negative ΔGbind accounts for its stable interaction with Omicron-RBD as compared to its mutant.

## 4. Conclusions

The RBD (Residues 331–524) of SARS-CoV-2 S glycoprotein constitutes an essential drug, vaccine, and mAb target due to its interaction with the human receptor ACE2, as well as its response in the host immune response [43]. Many mutations in the emerging viral variants are located in the RBD, which are targeted by a majority of human-neutralizing mAbs from COVID-19 patients and other mAb therapies under investigation [44]. The SARS-CoV-2 variants in the RBD interfere with the host immune system and impair the antibody-mediated neutralization of the virus. A majority of mAbs exhibit neutralizing activity against SARS-CoV-2, generated by blocking the attachment of the RBD to the hACE2 receptor [45]. However, due to the emergence of several new variants in the RBD during the pandemic, these mAbs showcase weak or reduced neutralization activities. Our MD simulation combined with MMGBSA study elucidates the effect of the RBD double mutation (L452R and E484Q) on the binding affinity of different mAbs that interact with the S protein. These mutations were found to alter the interfacial interactions between the spike RBD and mAb. REGN10933, BD368-2 (Class II), S2M11 (Class III), and JMB2002 (Class V) were found to display better binding affinity with WT-RBD as compared to MT-RBD. This can be attributed to the comparatively more stable hydrophobic, electrostatic, and hydrogen bond interactions in the WT complex as compared to MT-RBD. The decrease in binding affinity of mutated complexes is majorly due to the occurrence of unfavorable interactions between the RBD and mAb. The observed double mutation can be associated with weakened neutralizing activity and immune evasion. EY6A mAb was observed to have greater binding affinity with MT-RBD than WT, indicating that these were also consistent in their action against a mutated virus. This study may provide useful information in the ongoing development of effective vaccines and therapeutic antibodies.

## Figures and Tables

**Table 1:** The crystal structure complexes of RBD and various monoclonal antibodies (mAb) taken for the study.

RBD Complex with mAb	PDB ID	Type of Class
RBD–REGN10933	6XDG	Class I
RBD–BD368-2	7CHF	Class II
RBD–S2M11	7K43	Class III
RBD–EY6A	6ZCZ	Class IV
RBD–JMB2002	7WRV	Class V

**Table 2:** The obtained RMSF values for WT and MT-RBD complex with mAb in Å.

RMSF Values of Residues of RBD	RBD REGN10933	RBD-BD-368-2	RBD EY6A	RBD S2M11	RBD JMB2002
L452	0.997	1.05	2.24	0.842	1.2
L452R	1.29	2.58	2.84	0.943	1.8
E484	2.0	0.981	6.421	1.85	-
E484Q	2.5	2.14	6.9	2.26	-

**Table 3:** The predicted binding free-energy calculations for both WT and MT-RBD complex with mAb. The units of the energies are in kcal/mol.

RBD Complex with Antibody	MMGBSA_ΔGBind	Coulomb	Covalent	Bind H Bond	Bind_Lipo	Bind_vdW	Solv_GB
RBD–REGN 10933 (WT)	−87.58 ± 3.2	−40.66 ± 2.5	−7.0 ± 0.05	−8.31 ± 0.65	−26.41 ± 0.16	−79.2 ± 8.2	74.24 ± 9.4
RBD–REGN 10933 (MT)	−76.29 ± 1.7	−29.28 ± 1.2	−13.31 ± 0.12	−9.21 ± 2.25	−28.18 ± 0.28	−108.34 ± 10.1	94.00 ± 7.0
RBD-BD- 368-2 (WT)	−65.43 ± 1.2	−89.94 ± 3.4	−2.8 ± 0.12	−5.3 ± 0.05	−21.84 ± 0.21	−98.01 ± 6.7	156.8 ± 16
RBD–BD-368-2 (MT)	−17.96 ± 1.2	−35.20 ± 2.8	−0.88 ± 0.03	−1.45 ± 0.02	−3.155 ± 0.03	−10.52 ± 0.24	41.04 ± 4.1
RBD–S2M11 (WT)	−78.68 ± 1.5	−39.58 ± 5.2	−6.51 ± 0.65	−1.85 ± 0.05	−33.27 ± 0.04	−107.95 ± 11.2	109.6 ± 14
RBD–S2M11 (MT)	−60.47 ± 0.09	−49.03 ± 16.0	2.27 ± 0.39	−1.80 ± 0.09	−35.21 ± 0.09	−90.3 ± 3.3	92.71 ± 17.2
RBD–EY6A (WT)	−88.23 ± 0.87	−106.05 ± 9.0	−5.85 ± 0.34	−4.43 ± 0.5	−12.40 ± 0.012	−59.8 ± 4.23	105.61 ± 11.6
RBD–EY6A (MT)	−96.53 ± 6.2	−74.42 ± 3.5	−17.95 ± 0.17	−5.96 ± 0.23	−18.63 ± 0.8	−78.41 ± 6.7	99.84 ± 9.8
Omicron-RBD–JMB2002	−82.68 ± 4.2	−96.53 ± 6.2	−5.53 ± 0.2	−4.43 ± 6.2	−12.53 ± 1.2	−78.53 ± 6.2	107.53 ± 6.2
Omicron (L452R)-RBD–JMB2002	−76.28 ± 3.2	−106.53 ± 6.2	−6.53 ± 0.2	−4.43 ± 6.2	−10.53 ± 1.2	−68.53 ± 6.2	112.53 ± 6.2

**Table 4:** The energy contribution of L452 and L452R and E484 and E484Q double-mutant residues in overall stabilization of both WT and MT–RBD complex with mAb.

	Residue	dG Bind	Coulomb	Solvation	vdW	H Bond	Lipo
RBD- REGN-10933	L452	−0.03	0.06	−0.1	−0.04	0	0
	L452R	−1.36	−10.11	9.08	−0.13	0.03	0
	E484	0.51	7.68	−7.49	−0.03	−0.42	0
	E484Q	−4.93	−4.95	4.33	−3.68	−0.41	−1.33
RBD-BD-368-2	L452	−1.17	0.87	−0.77	−1.54	0	−0.69
	L452R	−0.9	−5.89	5.46	−0.52	−0.09	−0.08
	E484	−5.34	−10.81	9.55	−3.73	−1.75	−0.76
	E484Q	−0.86	−1.77	1.92	−1.1	−0.24	−0.09
RBD-S2M11	L452	−2.57	−16.99	15.02	−1.68	−0.83	−0.24
	L452R	−0.86	−10.77	10.27	−0.4	−0.08	−0.04
	E484	−2.83	−2.92	3.88	−3.57	−0.21	−0.56
	E484Q	−2.63	−2.77	−1.1	−2.17	−0.33	−0.15
RBD-EY6A	L452	−0.01	−0.14	0.23	−0.01	0	0
	L452R	0.04	−6.98	6.62	−0.02	−0.03	0
	E484	−0.08	3.15	−3.35	0.01	0	0
	E484Q	0.08	−0.05	0.06	−0.02	0	0
Omicron L452 RBD–L452R JMB2002		−2.19	−0.21	−0.1	−1.94	0	0
		−0.76	−0.14	1.0	−1.54	0.01	0
					

## Data Availability

Dataset used for this study is available from D.G. on request.

## Supplementary Materials

The following supporting information can be downloaded at: https://www.mdpi.com/article/10.3390/vaccines11010023/s1, Figure S1 (A): Cartoon representation of wild type (WT)–receptor-binding domain (RBD) with EY6A monoclonal antibody (mAb). (B) Cartoon representation of mutant type (MT)–receptor-binding domain (RBD) with EY6A monoclonal antibody (mAb).
