# Supplementary material for: Effect of Double Mutation (L452R and E484Q) on the Binding Affinity of Monoclonal Antibodies (mAbs) against the RBD—A Target for Vaccine Development
Source: Vaccines (Basel). 2022 Dec 22;11(1):23. doi: 10.3390/vaccines11010023 (PMC9860914; doi:10.3390/vaccines11010023)
Supplement: Supplementary file 1 [file vaccines-11-00023-s001.zip › vaccines-2071511-supplementary.pdf]

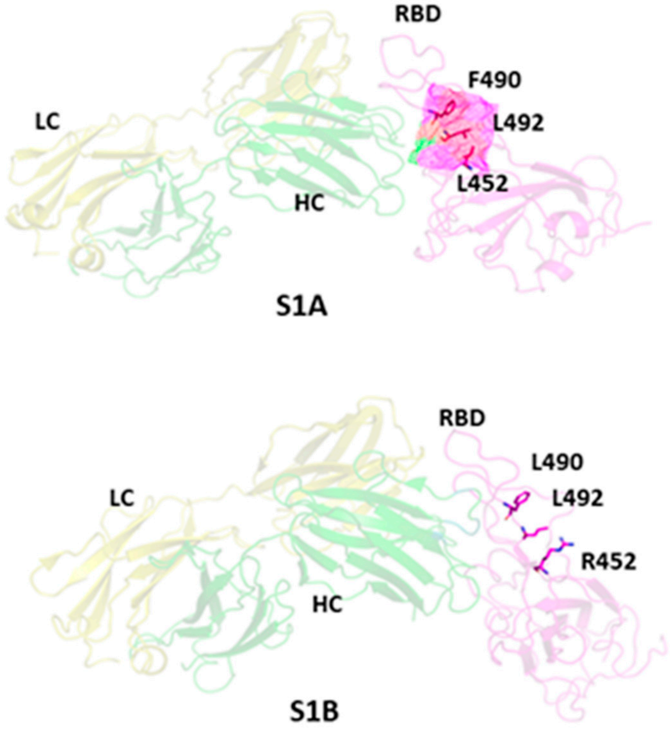

Figure S1. (**A**) **Cartoon representation of wild type (WT)–receptor-binding domain (RBD) with EY6A monoclonal antibody (mAb).** L452 of RBD forms intramolecular hydrophobic patch with L492 and F490 residues. Magenta color represents RBD, while green and yellow colors represent heavy chain (HC) and light chain (LC) of EY6A. (**B**) **Cartoon representation of mutant type (MT)–receptor-binding domain (RBD) with EY6A monoclonal antibody (mAb).** In the mutant, presence of charged R452 of RBD abolishes the intramolecular hydrophobic patch with L492 and F490 residues. Magenta color represents RBD, while green and yellow colors represent heavy chain (HC) and light chain (LC) of EY6A.
